# Norfloxacin and N-Donor Mixed-Ligand Copper(II) Complexes: Synthesis, Albumin Interaction, and Anti-*Trypanosoma cruzi* Activity

**DOI:** 10.1155/2016/5027404

**Published:** 2016-01-26

**Authors:** Darliane A. Martins, Ligiane R. Gouvea, Gabriel S. Vignoli Muniz, Sonia R. W. Louro, Denise da Gama Jaen Batista, Maria de Nazaré C. Soeiro, Letícia R. Teixeira

**Affiliations:** ^1^Departamento de Química, Universidade Federal de Minas Gerais, 31270-901 Belo Horizonte, MG, Brazil; ^2^Departamento de Física, Pontifícia Universidade Católica do Rio de Janeiro, 22653-900 Rio de Janeiro, RJ, Brazil; ^3^Laboratório de Biologia Celular, Instituto Oswaldo Cruz, FIOCRUZ, 21040-360 Rio de Janeiro, RJ, Brazil

## Abstract

Copper(II) complexes with the first-generation quinolone antibacterial agent norfloxacin containing a nitrogen donor heterocyclic ligand 2,2′-bipyridine (bipy) or 1,10-phenanthroline (phen) were prepared and characterized by IR, EPR spectra, molar conductivity, and elemental analyses. The experimental data suggest that norfloxacin was coordinated to copper(II) through the carboxylato and ketone oxygen atoms. The interaction of the copper(II) complexes with bovine serum albumin (BSA) and human serum albumin (HSA) was investigated using fluorescence quenching of the tryptophan residues and copper(II) EPR spectroscopy. The results of fluorescence titration revealed that copper(II) complexes have a moderate ability to quench the intrinsic fluorescence of the albumins through a static quenching mechanism. EPR experiments showed that BSA and HSA Cu(II) sites compete with NOR for Cu(II)-bipy and Cu(II)-phen to form protein mixed-ligand complexes. Copper(II) complexes, together with the corresponding ligands, were evaluated for their trypanocidal activity* in vitro* against* Trypanosoma cruzi*, the causative agent of Chagas disease. The tests performed using bloodstream trypomastigotes showed that the Cu(II)-N-donor precursors and the metal complexes were more active than the free fluoroquinolone.

## 1. Introduction

Chagas disease, caused by the* Trypanosoma cruzi* protozoa, is an endemic parasitosis which affects about 8 million people in Latin America and leads to approximately 50000 deaths per year [1]. The only two available drugs for the treatment of Chagas disease are Nifurtimox and Benznidazole, but both exhibit grave side effects [2]. The limitations of the current chemotherapy for this illness justify the search for new drug candidates that could be effective and selective against this parasite but with lower toxicity and affordable costs.

Norfloxacin (NOR), 1-ethyl-6-fluoro-1,4-dihydro-4-oxo-7-(1-piperazinyl)-quinoline-3-carboxylic acid, which was patented in 1978, is a synthetic and potent fluoroquinolone antibacterial agent [3]. NOR is used in the clinical treatment of many infections including prostate, skin, pulmonary, digestive, and urinary tract infections [4].

Metal complexes can be more active than their free ligands. Among the metal complexes so far investigated those containing N-donor heterocyclic coligand have attracted a great deal of attention. Ligands like 2,2′-bipyridine (bipy) and 1,10-phenanthroline (phen) coordinated to the metal center in the bidentate mode have been proven to be more active than monodentate ligands like pyridine against microorganisms. Considering the nature of the N-donor heterocyclic ligand the results found in the literature suggest that the inhibition of the growth of microorganisms increases in the order phen > bipy [5, 6].

In the literature there are some examples of norfloxacin copper(II) complexes which contain nitrogen donor ligands [6–11]. However, few examples concerning the anti-*T. cruzi *activity of fluoroquinolone complexes can be found [12–15] as well as their albumins interaction studies using EPR.

In a recent research, our group studied the anti-*T. cruzi* activity of the sparfloxacin and levofloxacin copper(II) complexes containing N-donor ligand and found that the presence of bipy and phen improves their biological activity. These complexes could bind to DNA, suggesting that the action mechanism could involve this molecule [12, 14].

In this work, we prepared two Cu(II)-norfloxacin complexes containing N-donor ligand, 2,2′-bipyridine or 1,10-phenanthroline, and their anti-*T. cruzi* activity was tested. Given that the interaction of a drug with blood components can influence its bioavailability [16], the interaction of these complexes with bovine and human serum albumins (BSA and HSA) was also investigated, using the intrinsic fluorescence of the proteins and the EPR spectroscopy of the copper(II) ions.

Albumin is the most abundant serum protein, representing 52–60% of total blood plasma proteins. Human serum albumin (HSA) binds different classes of ligands at multiple sites. HSA provides a storage area for many compounds, affects pharmacokinetics of many drugs, restrains the orientation of some ligands providing metabolic modification, makes potential toxins nontoxic transporting them to disposal sites, accounts for most of the antioxidant capacity of human serum, and acts as NO-carrier [17].

Until recently, X-ray structural investigations of mammalian serum albumins have only concentrated on human serum albumin (HSA) (structures deposited in http://www.rcsb.org/), but in 2012 the crystal structures of serum albumins isolated from bovine, equine, and leporine blood plasma were reported to be deposited in RBSC PDB (http://www.rcsb.org/) [18]. BSA is one of the most extensively used proteins in protein research and is used as HSA substitute in many experiments, but it exhibits only 75.8% identity compared with HSA [18]. The HSA has two major binding regions, sites I and II, 585 amino acid residues, and only one tryptophan (Trp) located at position 214 in a hydrophobic pocket. BSA has two tryptophan residues (Trp 134 and Trp 212), with Trp 134 being located on the surface of the molecule and Trp 212 being located in a hydrophobic pocket [16].

The tryptophan's intrinsic fluorescence has been extensively used to study the interaction between drugs and proteins [9, 13, 16, 19]. It is important, however, to have in mind that some binding sites are far from the Trp residues and no interaction will be detected. On the other hand, many drugs absorb radiation in the UV region of the electromagnetic spectrum and give rise to artifacts such as the inner filter effect, rather than quenching, and direct excitation of the fluorescence of certain drugs, rather than energy transfer. For this reason fluorescence quenching and energy transfer experiments must always follow optical absorption measurements, and careful corrections must be made.

## 2. Experimental

### 2.1. Materials

The BSA, HSA, norfloxacin, 2,2′-bipiridine, CuCl_2_·2H_2_O, and [CuCl_2_(phen)] were purchased from Sigma-Aldrich. All solvents were purchased from Merck.

4 × 10^−6^ mol L^−1^ BSA and HSA solutions were prepared in phosphate buffer at pH 7.4. 1.0 × 10^−3^ mol L^−1^ stock solution of the Cu(II) complexes was prepared using 2.5% of dimethylsulfoxide (DMSO) and phosphate buffer at pH 7.4.

### 2.2. Apparatus

Elemental analyses were performed on a PerkinElmer 2004 CHN Elemental Analyzer. Molar conductivity measurements were performed in dimethylformamide (DMF) solutions, 1 × 10^−3^ mol L^−1^ concentration, using a Quimis, model* Q405M*, conductivity meter. The IR spectra were acquired on a Mattson Instruments Galaxy 3000 spectrophotometer using KBr pellets. X-band electron paramagnetic resonance (EPR) spectra were obtained with a Bruker ESP300E spectrometer with a modulation frequency of 100 kHz and a modulation amplitude of 1 mT. Frozen aqueous solutions of the complexes (~5 × 10^−4^ mol L^−1^) were measured at liquid N_2_ temperature (77 K) in Teflon^*®*^ tubes with a 3 mm internal diameter.

### 2.3. Synthesis of the [CuCl_2_(bipy)] Precursor

The [CuCl_2_(bipy)] precursor was prepared using a method similar to that described in the literature [14], by dissolving equimolar amounts of CuCl_2_·2H_2_O and 2,2′-bipyridine (1.74 mmol) in about 20 mL of acetone. The mixture was allowed to stir and reflux for 24 hours and then was vacuum filtered. [CuCl_2_(bipy)] was subjected to elemental and infrared analysis.

### 2.4. Synthesis of the Complexes

**Figure 1:**
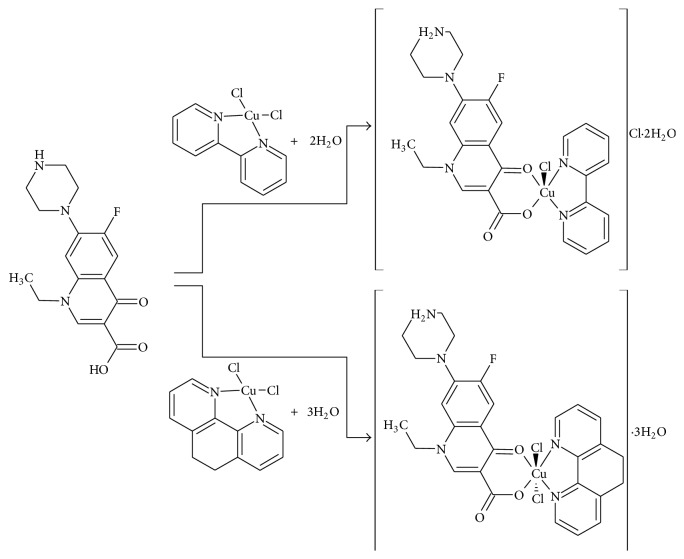
Coordination scheme for complexes** 1** and** 2**.

[CuCl(NOR)(bipy)]Cl (**1**) was obtained by dissolving NOR (0.31 mmol) in acetone (30 mL) which was gently heated and stirred. After cooling the solution to room temperature acetone solution of [CuCl_2_(bipy)] (0.31 mmol) was added. The mixture was stirred at room temperature for 24 h. The solid which precipitated was filtered and washed with diethyl ether and dried* in vacuum.* [CuCl_2_(phen)(NOR)] (**2**) was prepared according to a similar method previously described by our group [12]. The complex was obtained by dissolving 0.31 mmol of NOR in approximately 40 mL of acetone. After that 0.31 mmol of [CuCl_2_(phen)] was dissolved in methanol which was added to the NOR solution. The mixture remained refluxing and stirring for about 24 hours. The solvent volume was reduced using a rotary evaporator. The precipitate formed was filtered under vacuum conditions, washed with diethyl ether, and dried.  Figure 1 shows a coordination scheme for complexes** 1** and** 2**.

 [CuCl(bipy)(NOR)]Cl·2H_2_O (**1**): Green solid. Yield: 73%. Anal.: found, C 48.2, H 4.5, N 10.8. Calc. for C_26_H_30_Cl_2_CuFN_5_O_5_, C 48.3, H 4.7, N 10.8%. Molar conductivity (1 × 10^−3^ mol L^−1^, H_2_O): 116.5 *μ*S cm^−1^. IR (cm^−1^): 1570 s *ν*(C=O)_*p*_; 1630 s *ν*_as_(COO^−^); 1396 m *ν*_*s*_(COO^−^). TG: mass loss (351–439 K): 10.5% (found), 11.0% (calc).
 [CuCl_2_(phen)(NOR)]·3H_2_O (**2**): Green solid. Yield: 90%. Anal.: found, C 48.9, H 4.6, N 10.4. Calc. for C_28_H_32_Cl_2_CuFN_5_O_6_, C 48.9, H 4.7, N 10.2%. Molar conductivity (1 × 10^−3^ mol L^−1^, DMF): 22.4 *μ*S cm^−1^. IR (cm^−1^): 1580 s *ν*(C=O)_*p*_; 1628 s *ν*_as_(COO^−^); 1386 m *ν*_*s*_(COO^−^). TG: mass loss (351–434 K): 7.0% (found), 7.8% (calc).

### 2.5. Parasites

Y strain of* T. cruzi *was used throughout the experiments [21]. Bloodstream forms were harvested by heart puncture from* T. cruzi*-infected Swiss mice at the peak of parasitemia [21].

### 2.6. Trypanocidal Analysis

For the* in vitro *analysis on trypomastigotes, the parasites were incubated at 310 K in the presence of increasing doses (0–200 *μ*M) of each compound diluted in Dulbecco's modified Eagle's medium which was supplemented with 5% fetal bovine serum and 1 mM* L*-glutamine (DMES) [22]. After 24 h, death rates were determined by light microscopy through the direct quantification of live parasites using Neubauer chamber, and EC_50_ values (drug concentration which reduces 50% of the number of live parasites) were then calculated as reported [2, 23].

### 2.7. Mammalian Cell Cultures and Toxicity Assays

Primary cultures of embryonic cardiac cells (CM) were carried out from mice embryos taken from pregnant females (18–20 days gestation). Pregnant animals were euthanized and the hearts' embryos collected. Then the ventricles were subjected to successive steps of mechanical and enzymatic dissociation (0.05% trypsin and 0.01% collagenase for 5 min/37°C). After purification, the CM were seeded at a density of 0.05 × 10^6^ cell/well into 96-well microplates, containing gelatin-coated cover slips and sustained in Dulbecco's modified Eagle's medium, which was supplemented with 5% fetal bovine serum, 2.5 mM CaCl_2_, 1 mM* L*-glutamine, and 2% chicken embryo extract (DMEM) [24]. All procedures were carried out in accordance with the guidelines established by the FIOCRUZ Committee of Ethics for the Use of Animals (License LW-16/14). All the cultures were maintained at 37°C in an atmosphere of 5% CO_2_, and the assays were run at least three times in duplicate. In order to rule out the toxic effects of the compounds on mammalian host cells, uninfected CMs were incubated for 24 h at 37°C in the presence or absence of the compounds (up to 200 *μ*M) diluted in DMEM, and then their morphology was evaluated by light microscopy and the cell viability was measured by the MTT colorimetric assay [25]. As control, only vehicle was used. The absorbance was measured at 490 nm wavelength with a spectrophotometer (VERSAmax Tunable, Molecular Devices, USA) allowing the determination of LC_50_ values (drug concentration which reduces 50% of cellular viability) and the respective selective indexes (SI = LC_50_/EC_50_).

### 2.8. Albumin Binding Studies

Steady state fluorescence measurements were performed on a Varian-Agilent Cary Eclipse or a PTI QM1 fluorescence system. UV-Vis absorption spectra were obtained with an Agilent diode array spectrophotometer model 8452A. Fluorescence lifetimes were measured using IBH-Horiba-Jobin Yvon TCSPC system. NanoLEDs with 1.0 ns nominal pulse duration and 1 MHz repetition rate were used as light sources for exciting the intrinsic HSA or BSA fluorescence (283 nm) and NOR fluorescence (330 nm).

Quenching measurements of albumin fluorescence were taken in 3 mL of BSA or HSA (4 × 10^−6^ mol L^−1^) in phosphate buffer 10 mM at pH 7.4. The albumin solutions were titrated by successive additions of the complex stock solutions. The fluorescence emission spectra of BSA and HSA were measured using an excitation wavelength of 290 nm. Experiments were performed at ambient temperature (296 K) and pH 7.4.

For the EPR studies of the interaction of the complexes with HSA and BSA, equimolar solutions (0.5 × 10^−3^ mol L^−1^) of BSA or HSA with each Cu(II) complex were prepared in 0.020 mol L^−1^ phosphate buffer at pH 7.4. EPR spectra were obtained at 77 K.

## 3. Results and Discussion

### 3.1. Microanalyses and Molar Conductivity Studies

Microanalyses and molar conductivity data suggested the formation of [CuCl(bipy)(NOR)]Cl·2H_2_O (**1**) and [CuCl_2_(phen)(NOR)]·3H_2_O (**2**), in which the fluoroquinolone NOR coordinated as a neutral bidentate ligand. The thermogravimetric data confirmed the presence of hydration water molecules in the complexes' structures. Complex** 1** exhibited a pentacoordinated structure in keeping with most examples found in the literature for Cu(II)-fluoroquinolone-N,N-donor complexes. These presented a square pyramidal geometry which was confirmed by X-ray crystallographic structures [7–10, 26]. Complex** 2** was hexacoordinated and presented an octahedral geometry which is not common for this type of complexes. In the literature there are examples of X-ray crystallographic structures for hexacoordinated fluoroquinolone complexes without N,N-donor [27, 28].

### 3.2. Infrared Spectral Studies

The infrared spectrum of free norfloxacin (NOR) exhibited a band of 1730 cm^−1^ which was assigned to the valence vibration of the carboxylic stretch *ν*(C=O)_carb_ and a band at 1616 cm^−1^ which was assigned to pyridone stretch *ν*(C=O)_*p*_ [29]. The most typical vibrations that were characteristic of the coordination type of quinolones were used in the metal-quinolone complex characterization. In the IR spectra of complexes** 1 **and** 2** the absorption of the *ν*(C=O)_carb_ vibration was not observed, due to the deprotonation of the carboxylic group, indicating that this group was involved in coordination. Two new very strong characteristic bands appeared at 1630 and 1628 cm^−1^ and 1396 and 1386 cm^−1^ and were assigned to *ν*(COO^−^) asymmetric and symmetric stretching vibrations for complexes** 1** and** 2**, respectively, whereas *ν*(C=O)_*p*_ was shifted from 1616 to 1570 and 1580 cm^−1^ upon coordination for** 1 **and** 2**, respectively.

The Δ = *ν*(COO^−^)_asym_ − *ν*(COO^−^)_sym_ difference is a useful characteristic for determining the coordination mode of the quinolone ligands. Δ values for complexes** 1 **and** 2 **were 234 and 242 cm^−1^, respectively, indicating a monodentate coordination mode of the NOR carboxylato group [30]. The overall changes of the IR spectra suggested that the norfloxacin ligand was coordinated to Cu(II) via the pyridone and one carboxylate oxygen in the neutral zwitterionic form.

### 3.3. EPR Spectra of the Copper Complexes

**Figure 2:**
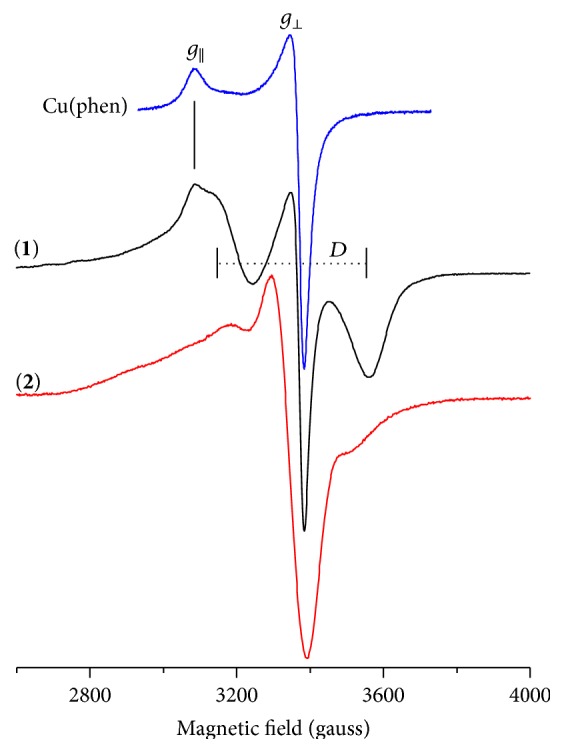
X-band EPR spectra of [CuCl_2_(phen)] and complexes** 1** and** 2 **(powder, ambient temperature).

Room temperature X-band EPR spectra of** 1** and** 2** complexes powder samples are presented in Figure 2 and the parameters are in Table 1. The spectrum of [CuCl_2_(phen)] is also presented for comparison, which is characteristic of mononuclear copper complexes with axial symmetry, and lacks the hyperfine splitting at room temperature, as commonly observed in concentrated solid Cu(II) complexes [14]. Complex** 1 **is a mixture of mononuclear and binuclear Cu(II) complexes. Its EPR spectrum is a superposition of spectrum similar to Cu(phen) spectrum and a well resolved doublet, associated with the triplet state (*S* = 1, Δ*m*_*S*_ = ±1) of binuclear complexes [14, 31, 32].

The room temperature EPR spectrum of complex** 2 **is a superposition of a very broad (~330 gauss) component due to strong dipole-dipole interactions superimposed to a 100 gauss line width component at the *g*_⊥_ region.

**Figure 3:**
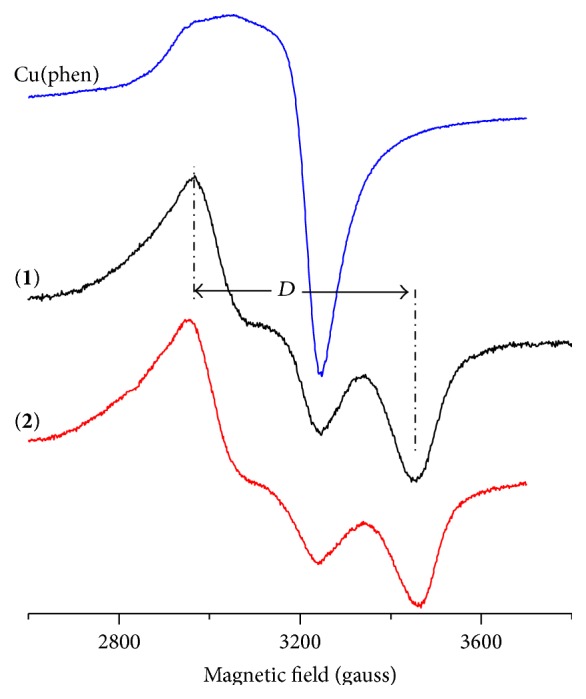
EPR spectra of [CuCl_2_(phen)] and complexes** 1** and** 2** in water (~10^−3^ mol L^−1^) at 77 K.

The EPR spectra of [CuCl_2_(phen)] and complexes** 1** and** 2** in water at 77 K are presented in Figure 3 (parameters are in Table 1). It can be observed that the spectra of the mixed-ligand complexes are almost exclusively due to binuclear species, with a small fraction of mononuclear species, while [CuCl_2_(phen)] presents a mononuclear spectrum where the absence of hyperfine splitting suggests aggregation leading to exchange [12].

The distance between the two Cu(II) ions can be estimated from the zero field splitting parameter *D*. The average distance *r* between the two coupled unpaired electrons can be calculated by using the following equation:

(1) $$\begin{array}{c} D=\frac{3}{2}\frac{g\beta }{r^{3}}=1.39\times 10^{4}\frac{g}{r^{3}}, \end{array}$$

for *D* in gauss and *r* is in angstroms [33]. The calculated distance *r* for the binuclear complexes** 1** and** 2 **is 3.9 Å. This distance is similar to those obtained for other Cu(II) binuclear complexes [12, 34, 35].

### 3.4. Anti-*Trypanosoma cruzi* Activity

The effect of all the complexes, precursors, and benznidazole, the reference drug, against bloodstream trypomastigote forms of* T. cruzi *(Y strain), expressed as EC_50_, and their corresponding selectivity index (SI, ratio between LC_50_ and EC_50_ values, Table 2) were evaluated.

The free norfloxacin (NOR) and CuCl_2_ exerted a low trypanocidal effect against bloodstream trypomastigotes, exhibiting an EC_50_ value of 126 ± 30 and 83 ± 3 *μ*M, respectively. The same could be observed when trypomastigotes were exposed to [CuCl_2_NOR], which showed an EC_50_ value of 78 ± 12 *μ*M (Table 2).

The complexation of Cu(II)-NOR to 2,2′-bipyridine (bipy) improves the anti-*T. cruzi *activity. [CuCl_2_(bipy)(NOR)] exhibited an EC_50_ value of 16 ± 4 *μ*M and the [CuCl_2_(bipy)] precursor exhibited an EC_50_ value of the same order: EC_50_ = 14 ± 7 *μ*M. Therefore we suggest that the activity presented by complex** 1** can be related to the [CuCl_2_(bipy)] precursor that showed similar activity to the reference drug, benznidazole (EC_50_ = 13 ± 2 *μ*M).

[CuCl_2_(phen)] and [CuCl_2_(phen)(NOR)] revealed themselves as the most active compounds, exhibiting EC_50_ values of 7 ± 5 and 4.4 ± 1.4 *μ*M, respectively. These compounds were 2-3 times more active than benznidazole, being the most promising anti-*T. cruzi* agents.

The relative toxicity of the free bases and their metal complexes was evaluated in uninfected CM. After 24 h of treatment, all compounds induced loss of cellular viability and cell contractility in doses >12 *μ*M (Table 2) and thus the corresponding low SI values were suggestive of a generic toxicity.

### 3.5. Albumin Binding Studies

In this section, the quenching of BSA and HSA fluorescence by Cu(II) ions, Cu(II)-bipy, Cu(II)-phen, and complexes** 1** and** 2 **was monitored. All the UV absorption and emission spectra were registered. The fluorescence decay curves with excitation at 283 nm and emission at 340 nm were obtained in the absence of the quenchers, at an intermediary and at the final quencher concentration. The raw data are available in Supplementary Material available online at http://dx.doi.org/10.1155/2016/5027404. The fluorescence intensity was attenuated by absorption of the incident and emitted light. These attenuation effects called primary and secondary inner filter effects, respectively, do not contain molecular information. The fluorescence spectra were corrected using the following expression [36]:

(2) $$\begin{array}{c} F_{corr}=F_{obs}10^{\left[\left(A_{ex}+A_{em}\right)l/2\right]}, \end{array}$$

where *F*_corr_ and *F*_obs_ are the corrected and observed fluorescence intensity, *A*_ex_ and *A*_em_ are the absorbance at the excitation and emission wavelengths, respectively, and *ℓ* is the optical path in cm. This expression assumes that the absorbing and emitting portion of the sample is localized at the center of the cuvette and was observed to be a good approximation for absorbances less than 0.5.

**Figure 4:**
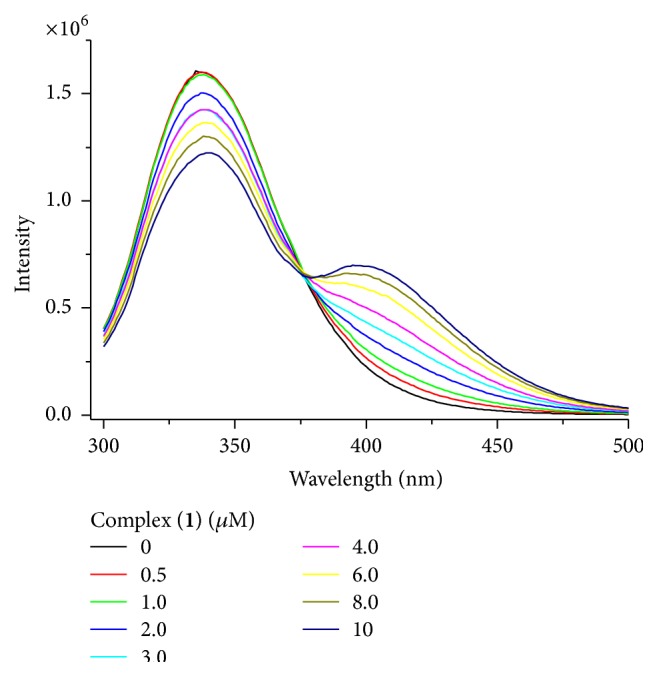
Fluorescence spectra of BSA (4 *μ*mol L^−1^) in the absence and presence of increasing amounts of [CuCl(bipy)(NOR)]Cl (**1**).

Figure 4 shows an example of corrected fluorescence spectra obtained by titrating BSA with complex** 1**, with excitation at 290 nm. BSA has a strong fluorescence emission peak at 338 nm due to the two Trp residues, while HSA (supplementary material) has a fluorescence emission peak at 336 nm due to the single Trp 214 residue. The fluorescence of tyrosine residues is negligible with the used excitation wavelength.

**Figure 5:**
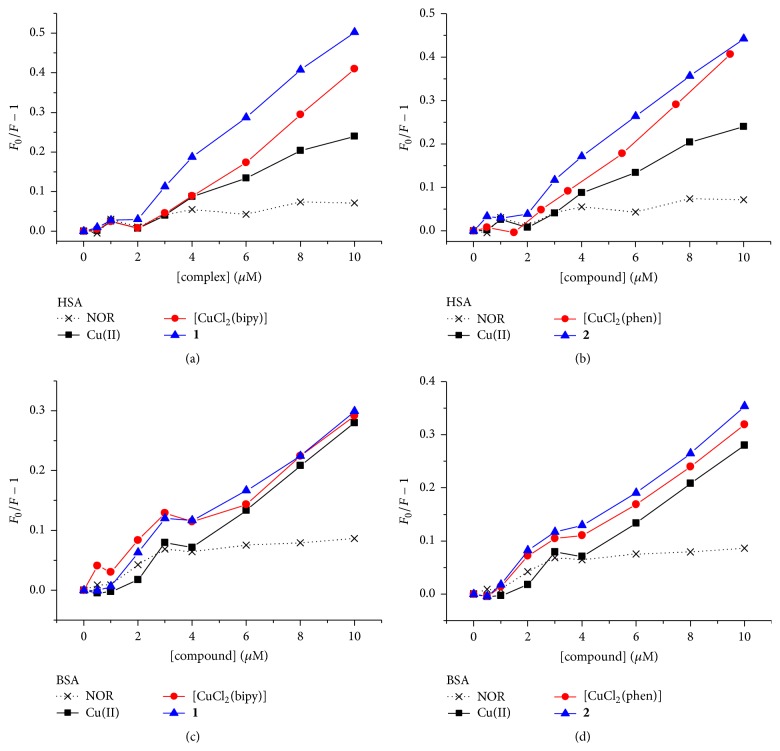
Stern-Volmer plots for the quenching of albumins' intrinsic fluorescence by the copper complexes: (a) HSA-complex (**1**), (b) HSA-complex (**2**), (c) BSA-complex (**1**), and (d) BSA-complex (**2**). Control results of the effects of NOR and Cu(II) are also presented. *F*_0_ and *F* are the fluorescence intensities at the peak in the absence and presence of the complexes. HSA and BSA concentrations equal to 4.0 × 10^−6^ mol L^−1^; temperature: 296 K.

The fluorescence intensity of BSA and HSA was observed to decrease in the presence of Cu(II) and all four complexes (Figure 5), indicating that Cu(II) is an important quenching unit. Figure 5 shows the Stern-Volmer plots for the quenching of HSA and BSA fluorescence by the complexes. It can be observed that at low concentrations there is no quenching, suggesting a high affinity site for Cu(II) and complexes which does not interact with the Trp residue(s). For complex concentrations greater than 2 or 4 × 10^−6^ mol L^−1^, the plots are linear, suggesting interaction at a second site.

Quenching of a fluorophore by a drug can occur by a static or dynamic process. Static or dynamic quenching at a single site can be described by the Stern-Volmer equation [36]:

(3) $$\begin{array}{c} \frac{F_{0}}{F}=1+K_{SV}\left[\;Q\;\right], \end{array}$$

where *F*_0_ and *F* are the fluorescence intensities of albumins in the absence and presence of complexes, respectively. [*Q*] is the quencher concentration, and *K*_SV_ is the Stern-Volmer quenching constant, which is related to the bimolecular collisional process in dynamic quenching but is the association constant in static quenching. Stern-Volmer constants were obtained for the linear region of the plots (Table 3).

The Stern-Volmer constant, *K*_SV_, was of the order of 10^4^ L mol^−1^ indicating that the complexes have a moderated interaction with HSA and BSA.

Both static quenching and dynamic quenching require molecular contact between the fluorophore and quencher. In the case of collisional quenching, the quencher must diffuse to the fluorophore during the lifetime of the excited state. Upon contact, the fluorophore returns to the ground state without emission of a photon and the lifetime of the excited state changes. In static quenching a nonfluorescent complex is formed between the fluorophore and the quencher, which does not contribute to fluorescence, and the lifetime does not change [36]. In order to distinguish static quenching from dynamic quenching, the fluorescence decays and lifetimes of HSA and BSA were measured by using the time-correlated single photon counting (TCSPC) technique. It was observed that the decay profile was not influenced by the presence of the Cu(II) ions or Cu(II) complexes. It is therefore concluded that a nonfluorescent ground state complex is formed.

Resonance energy transfer (RET) also reduces the fluorescence intensity and differs from static or dynamic quenching because it does not require molecular contact. The drug must be an acceptor for the donor molecule (Trp residue, in this case); that is, the absorption spectrum of the drug must overlap with the Trp emission spectrum. NOR is a good candidate for Trp acceptor (see absorption spectrum in supplementary material), but it was observed that the fluorescence increase around 408 nm (see Figure 4) has the same magnitude as the direct excitation of NOR. Furthermore, the absence of lifetime modifications eliminates the suggestion of RET.

Živec et al. [9] found that 1 : 2 Cu : NOR and 1 : 1 : 1 Cu : NOR : phen complexes exhibit good binding propensity to human or bovine serum albumin with binding constants of the same order of magnitude as these obtained by us and shown in Table 3. However, they did not correct their quenching results for the inner filter effect and their binding constants are overestimated.

### 3.6. Albumin Binding of the Cu(II) Complexes: EPR Studies

The human and bovine serum albumins have at least two Cu(II) binding sites (denoted as Cu_(1)_ and Cu_(2)_) including a strong N-terminal site that binds Cu(II) in a square-planar geometry via four nitrogen ligands (Cu_(1)_), which is similar in both human and bovine albumins [37–39]. This highest affinity binding site for the metal is formed by *α*-NH_2_ N atom, N atoms of the first two peptide bonds, and N_3_ atom of His3. This N-terminal site is fairly specific for transition metal ions and binds Cu(II) and Ni(II) in forms that will not generate reactive species [17].

Cys34 is the only cysteine with a free sulfhydryl group which does not participate in a disulfide linkage with any external ligand [40] and is involved in the second copper binding site. There is evidence that the second Cu(II) binds to deprotonated Cys34 residue [41].

**Figure 6:**
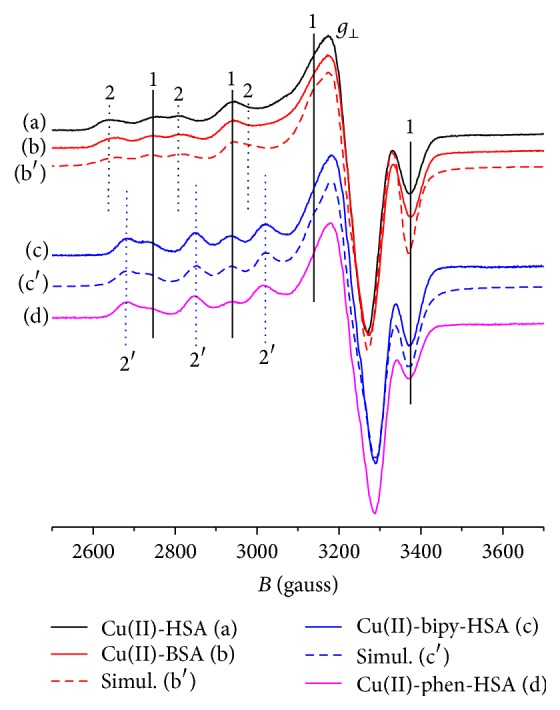
X-band EPR spectra for HSA and BSA Cu(II) complexes ((a) and (b), resp.) and for Cu(II)-bipy and Cu(II)-phen complexes with HSA ((c) and (d), resp.). Experimental conditions: Cu, HSA, and BSA 0.5 mM, phosphate buffer 20 mM, and pH 7.4, at 77 K. (b′) and (c′) are the simulated spectra of (b) and (c), using EasySpin [20] with parameters in Table 4.

Figure 6 displays the EPR spectra of Cu(II) (0.5 mM) in the presence of equimolar amounts of HSA (a) and BSA (b). The spectra are characteristic of a superposition of two binding sites (hyperfine lines labeled 1 and 2 in the *g*_||_ region, in Figure 6). The *g*_⊥_ region is a superposition of the lines from both sites. It can be noticed that the HSA and BSA spectra are very similar. At pH 7.4 both sites are occupied even at a 1 : 1 molar ratio, as already found by Patel and Pandeya [42]. The Cu(II)-BSA EPR spectrum was simulated (b′) using EasySpin [20] and the parameters appear in Table 4.

**Figure 7:**
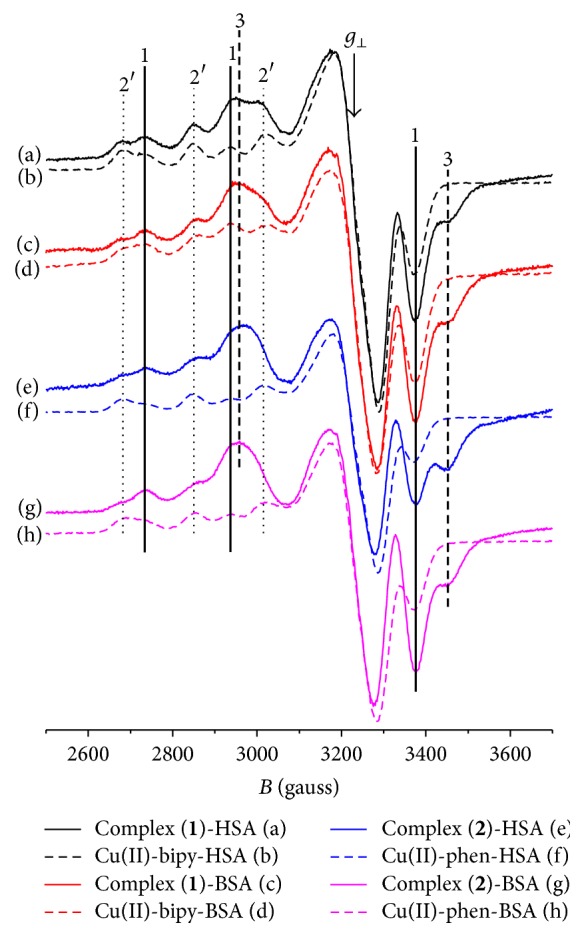
X-band EPR spectra for complexes** 1**, Cu(II)-bipy,** 2,** and Cu(II)-phen with equimolar amounts of HSA or BSA (concentration of the complexes: 0.5 mM, phosphate buffer 20 mM, pH 7.4, and temperature 77 K). (a)** 1**:HSA; (b) Cu(II)-bipy:HSA; (c)** 1**:BSA; (d) Cu(II)-bipy:BSA; (e)** 2**:HSA; (f) Cu(II)-phen:HSA; (g)** 2**:BSA; (h) Cu(II)-phen:BSA.

Figure 6 also shows the Cu(II)-bipy and Cu(II)-phen EPR spectra in the presence of equimolar amounts of HSA ((c) and (d)). The spectra of both complexes are very similar and also show a superposition of two binding sites. It can be noticed that the lines labeled 1 have the same positions in (c) and (d) as in (a) and (b), suggesting the same binding environment. This can be due to the displacement of the bipyridine and phenanthroline ligands by HSA to bind Cu(II) at the first site. The hyperfine lines of the second site (labeled 2′), however, are displaced to higher field values, indicating a *g*_||_ shift to a lower value relative to Cu(II) site 2. The EPR spectrum of Cu(bipy)(HSA) was also simulated (c′) using EasySpin [20] and the parameters for the two sites appear in Table 4. This analysis suggests formation of mixed-ligand complexes Cu(bipy)(HSA) and Cu(phen)(HSA) at this second site. The spectra ((c) and (d)) also show an increased population of site 2′ relative to site 1, indicating a higher affinity of this site for the complexes rather than for the naked Cu(II) ions. Similar results were found for BSA (see Figure 7, (d) and (h)).

Figure 7 shows the EPR spectra obtained when HSA and BSA are added to solutions of the copper complexes at a molar ratio of 1 : 1 ((a), (c), (e), and (g)). It can be observed that a fraction of the binuclear complexes (lines labeled 3) dissociates and binds to the albumins as mononuclear complexes, presenting EPR spectra characteristic of a superposition of two Cu(II) binding sites (lines labeled 1 and 2′). Figure 7 also shows the respective EPR spectra of Cu(II)-bipy and Cu(II)-phen with HSA and BSA added at a 1 : 1 molar ratio ((b), (d), (f), and (h)). The lines of binuclear complexes (labeled 3) are absent in the absence of NOR, but the lines for the two sites appear at the same positions (see vertical lines labeled 1 and 2′) and have therefore similar EPR parameters. It is concluded that both Cu(II) sites in albumin compete with NOR for the Cu(II)-bipy and Cu(II)-phen complexes. Probably, mixed-ligand complexes Cu(bipy)(SA) and Cu(phen)(SA) are formed at site 2, where SA stands for serum albumin. Site 1 can probably displace the two ligands, since spectral parameters are the same as for Cu(II) ion.

Comparing every two complexes' spectra with and without NOR (Figure 7), it is noticed that components 1 and 2′ appear with different fractions. Site 1 component is greater in presence ((a), (c), (e), and (g)) than in absence of NOR ((b), (d), (f), and (h)). The presence of NOR shifts the equilibrium toward a larger population of site 1. A possible explanation is that dissociated NOR molecules also bind to albumin near site 2 and compete with the complexes for this site.

Since the albumin molecules are not able to displace all the NOR molecules from the binuclear mixed-ligand complexes, one concludes that the association constants of both Cu(II)-bipy and Cu(II)-phen with the serum albumins are of the same order of magnitude as with NOR.

In the case of* T. cruzi *activity, similar results were observed for single and mixed-ligand complexes (see Table 2). This result suggests that Cu(II)-bipy and Cu(II)-phen were the main active species in these experiments. Based on the results demonstrating that the BSA and HSA Cu(II) sites compete with NOR for Cu(II)-bipy and Cu(II)-phen to form mixed-ligand complexes, it is reasonable to suggest that the important activity sites in* T. cruzi* can also displace NOR from the mixed complexes so that only Cu(II)-bipy and Cu(II)-phen bind to the relevant sites.

## 4. Conclusions

Two copper(II) complexes with norfloxacin in the presence of nitrogen donor heterocyclic ligand 2,2′-bipyridine or 1,10-phenantroline were obtained: the square pyramidal or trigonal bipyramidal [CuCl(bipy)(NOR)]Cl·2H_2_O (**1**) and the octahedral [CuCl_2_(phen)(NOR)]·3H_2_O (**2**). In these complexes, norfloxacin is coordinated to Cu(II) through the carboxylato and ketone oxygen atoms.

The trypanocidal activity of the copper complexes was tested using bloodstream trypomastigotes. It was shown that [CuCl_2_(phen)] and [CuCl_2_(phen)(NOR)]·3H_2_O were the most active compounds. However, EPR interaction studies showed that BSA and HSA compete with NOR to form mixed-ligand complexes, and only Cu(II)-phen binds to the relevant sites in these proteins. This suggests that Cu(II)-phen was responsible for the anti-*T. cruzi* activity.

The fluorescence titration results revealed that low concentrations of the Cu(II) complexes do not interact with BSA and HSA near the Trp residues. In fact, the highest affinity copper site in BSA and HSA involves the first three residues at the N-terminal, which is 31 to 35 Å away from Trp214 in HSA (4LA0 in RCSB PDB) or Trp213 in BSA (4F5S in RCSB PDB). The distance between the *α*-carbons of Asp1 and Trp134 in BSA is even larger, 40 Å. However, in concentrations greater than about half of the albumins concentration, the interaction occurs at the albumins' second site. *K*_SV_ values, about 10^4^ L mol^−1^, indicated a moderated affinity of this albumin site for the complexes. The EPR results suggest that this site displaces NOR from the complex and only Cu(II)-bipy and Cu(II)-phen bind to the protein.

EPR studies of the albumin binding to the Cu(II) complexes showed that the association constants of both Cu(II)-bipy and Cu(II)-phen with the serum albumins were of the same order of magnitude as with NOR, since the albumin molecules were not able to displace all the NOR molecules from the binuclear mixed-ligand complexes.

## Supplementary Material

The following Supplementary Material presents the data used in Albumin Binding Studies (section 3.5). The UV absorption and fluorescence emission spectra of BSA and HSA titrated with Cu(II) and the copper complexes are presented in Fig. S1 for BSA, and Fig. S2 for HSA. Fig. S3 presents an example of the inner filter effect correction described in the text and made on all the fluorescence spectra of Figs. S1 and S2. Finally, the corrected fluorescence spectra are presented in Fig. S4, for BSA, and Fig. S5 for HSA. These were used to obtain the data presented in Fig. 5.

## Figures and Tables

**Table 1:** EPR parameters of the Cu(II) complexes.

Powder (room temperature)	*g*_⊥_	*g*_||_	*g*_binuc_	*D* (gauss)
[CuCl_2_(phen)]	2.074	2.292		
[CuCl(bipy)(NOR)]Cl·2H_2_O (**1**)	2.068	2.250	2.08	425
[CuCl_2_(phen)(NOR)]·3H_2_O (**2**)	2.08			

Aqueous solution (77 K)	*g*_⊥_	*g*_||_	*g*_binuc_	*D* (gauss)

[CuCl_2_(phen)]	2.08	2.22		
[CuCl(bipy)(NOR)]Cl·2H_2_O (**1**)			2.08	490
[CuCl_2_(phen)(NOR)]·3H_2_O (**2**)			2.07	505

**Table 2:** Activity (mean ± SD) and selectivity index (SI) of the compounds and benznidazole upon bloodstream trypomastigotes (BT) forms of *T*. *cruzi* (Y strain) *in vitro* (24 h of incubation at 37°C).

Compound	EC_50_ (*μ*M)	SI
Norfloxacin (NOR)	126 ± 30	>1.25
[CuCl(bipy)(NOR)]Cl·2H_2_O (**1**)	16 ± 4	4
[CuCl_2_(phen)(NOR)]·3H_2_O (**2**)	4.4 ± 1.4	2.68
[CuCl_2_(bipy)]^*∗*^	14 ± 7	4.5
[CuCl_2_(phen)]^*∗*^	7 ± 5	<4
[CuCl_2_(NOR)]	78 ± 12	0.8
CuCl_2_^*∗*^	83 ± 3	6
Benznidazole	13 ± 2	77

SD: standard deviation of multiple experimental measurements.

SI: selective index: ratio between LC_50_/EC_50_ values.

LC_50_: drug concentration which reduces the viability of mammalian cell by 50%.

EC_50_: drug concentration which reduces the number of the parasites by 50%.

^*∗*^Data published in Martins et al., 2012 [14].

**Table 3:** Stern-Volmer constants values, *K*_SV_, for the titration of HSA and BSA with the copper complexes at 296 K (standard error, 0.2 × 10^4^ L mol^−1^).

Compound	HSA		BSA	
	*K*_SV_ (L mol^−1^)	*R* ^2^	*K*_SV_ (L mol^−1^)	*R* ^2^
Cu(II)	2.9 × 10^4^	0.998	3.5 × 10^4^	0.998
[CuCl_2_(bipy)]	5.0 × 10^4^	0.955	3.1 × 10^4^	0.955
[CuCl(bipy)(NOR)]Cl·2H_2_O (**1**)	5.8 × 10^4^	0.987	3.0 × 10^4^	0.987
[CuCl_2_(phen)]	5.1 × 10^4^	0.993	3.5 × 10^4^	0.993
[CuCl_2_(phen)(NOR)]·3H_2_O (**2**)	4.9 × 10^4^	0.989	3.7 × 10^4^	0.989

**Table 4:** EPR parameters obtained from simulated Cu(II) complexes interaction with HSA and BSA.

	Site 1		
	*g*_⊥_	*g*_||_	*A*_||_ (MHz)
Cu(BSA)	2.046	2.190	601 (196 G)
Cu(bipy)(HSA)	2.052	2.190	603 (197 G)

	Sites 2 and 2′		
	*g*_⊥_	*g*_||_	*A*_||_ (MHz)

[CuCl_2_(phen)]	2.074	2.292	479 (155 G)
Cu(BSA) site 2	2.070	2.299	505 (157 G)
Cu(bipy)(HSA) site 2′	2.062	2.265	528 (167 G)
