# Supplementary material for: Norfloxacin and N-Donor Mixed-Ligand Copper(II) Complexes: Synthesis, Albumin Interaction, and Anti-Trypanosoma cruzi Activity
Source: Bioinorg Chem Appl. 2016 Jan 26;2016:5027404. doi: 10.1155/2016/5027404 (PMC4746275; doi:10.1155/2016/5027404)
Supplement: Supplementary file 1 — The following Supplementary Material presents the data used in Albumin Binding Studies (section 3.5). The UV absorption and fluorescence emission spectra of BSA and HSA titrated with Cu(II) and the copper complexes are presented in Fig. S1 for BSA, and Fig. S2 for HSA. Fig. S3 presents an example of the inner filter effect correction described in the text and made on all the fluorescence spectra of Figs. S1 and S2. Finally, the corrected fluorescence spectra are presented in Fig. S4, for BSA, and Fig. S5 for HSA. These were used to obtain the data presented in Fig. 5. [file 5027404.f1.pdf]

Norfloxacin and N-donor mixed-ligand copper(II) complexes: synthesis, albumin interaction and anti-*Trypanosoma cruzi* activity.

Darliane A. Martins[a], Ligiane R. Gouvea[a], Gabriel S. Vignoli Muniz[b], Sonia R. W. Louro[b], Denise da Gama Jean Batista[c], Maria de Nazaré C. Soeiro[c] and Letícia R. Teixeira[a*]

[a]*Departamento de Química, Universidade Federal de Minas Gerais, 31270-901, Belo Horizonte (MG), Brazil.*

[b]*Departamento de Física, Pontifícia Universidade Católica do Rio de Janeiro, 22653-900, Rio de Janeiro (RJ), Brazil.*

[c]*Laboratório de Biologia Celular, Instituto Oswaldo Cruz, FIOCRUZ, 21040-360, Rio de Janeiro (RJ), Brazil.*

Supplementary Material

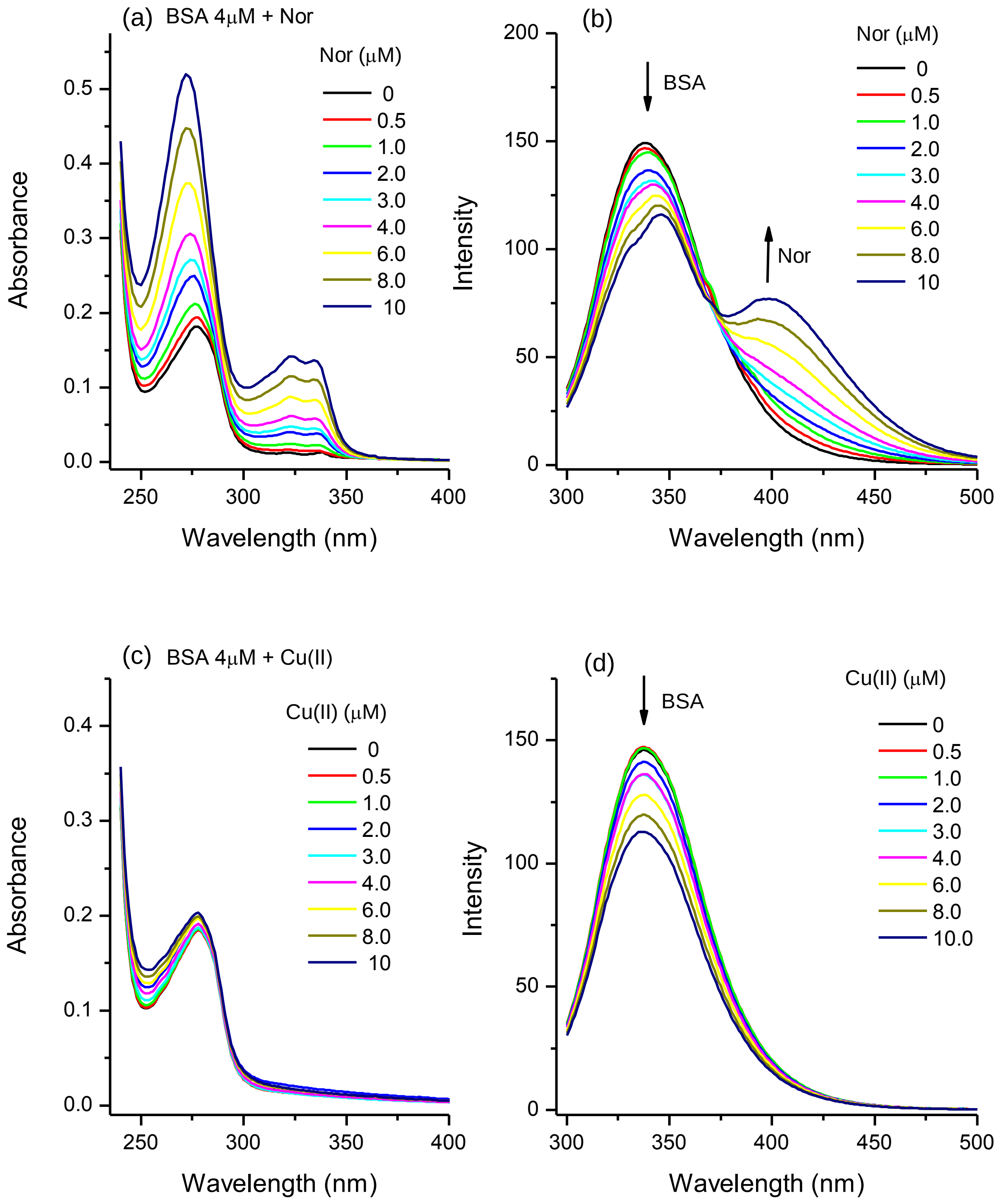
(a) BSA 4μM + Nor
Nor (μM)
0
0.5
1.0
2.0
3.0
4.0
6.0
8.0
10
Absorbance
Wavelength (nm)
(b)
Nor (μM)
0
0.5
1.0
2.0
3.0
4.0
6.0
8.0
10
BSA
Nor
Intensity
Wavelength (nm)
(c) BSA 4μM + Cu(II)
Cu(II) (μM)
0
0.5
1.0
2.0
3.0
4.0
6.0
8.0
10
Absorbance
Wavelength (nm)
(d)
BSA
Cu(II) (μM)
0
0.5
1.0
2.0
3.0
4.0
6.0
8.0
10.0
Intensity
Wavelength (nm)

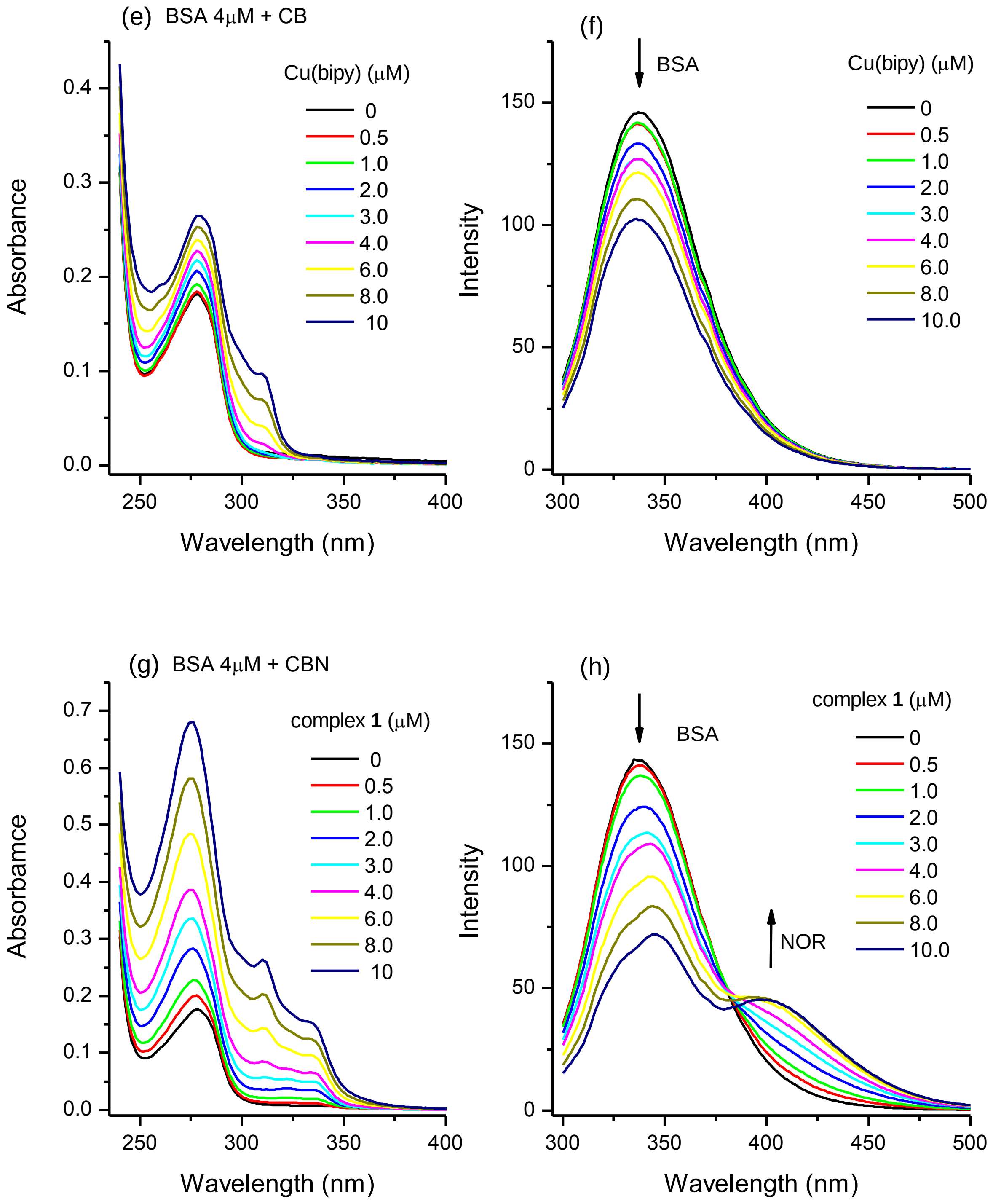

(e) BSA 4μM + CB
Cu(bipy) (μM)
0
0.5
1.0
2.0
3.0
4.0
6.0
8.0
10
Absorbance
Wavelength (nm)
(f)
BSA
Cu(bipy) (μM)
0
0.5
1.0
2.0
3.0
4.0
6.0
8.0
10.0
Intensity
Wavelength (nm)
(g) BSA 4μM + CBN
complex 1 (μM)
0
0.5
1.0
2.0
3.0
4.0
6.0
8.0
10
Absorbamce
Wavelength (nm)
(h)
BSA
complex 1 (μM)
0
0.5
1.0
2.0
3.0
4.0
6.0
8.0
10.0
NOR
Intensity
Wavelength (nm)

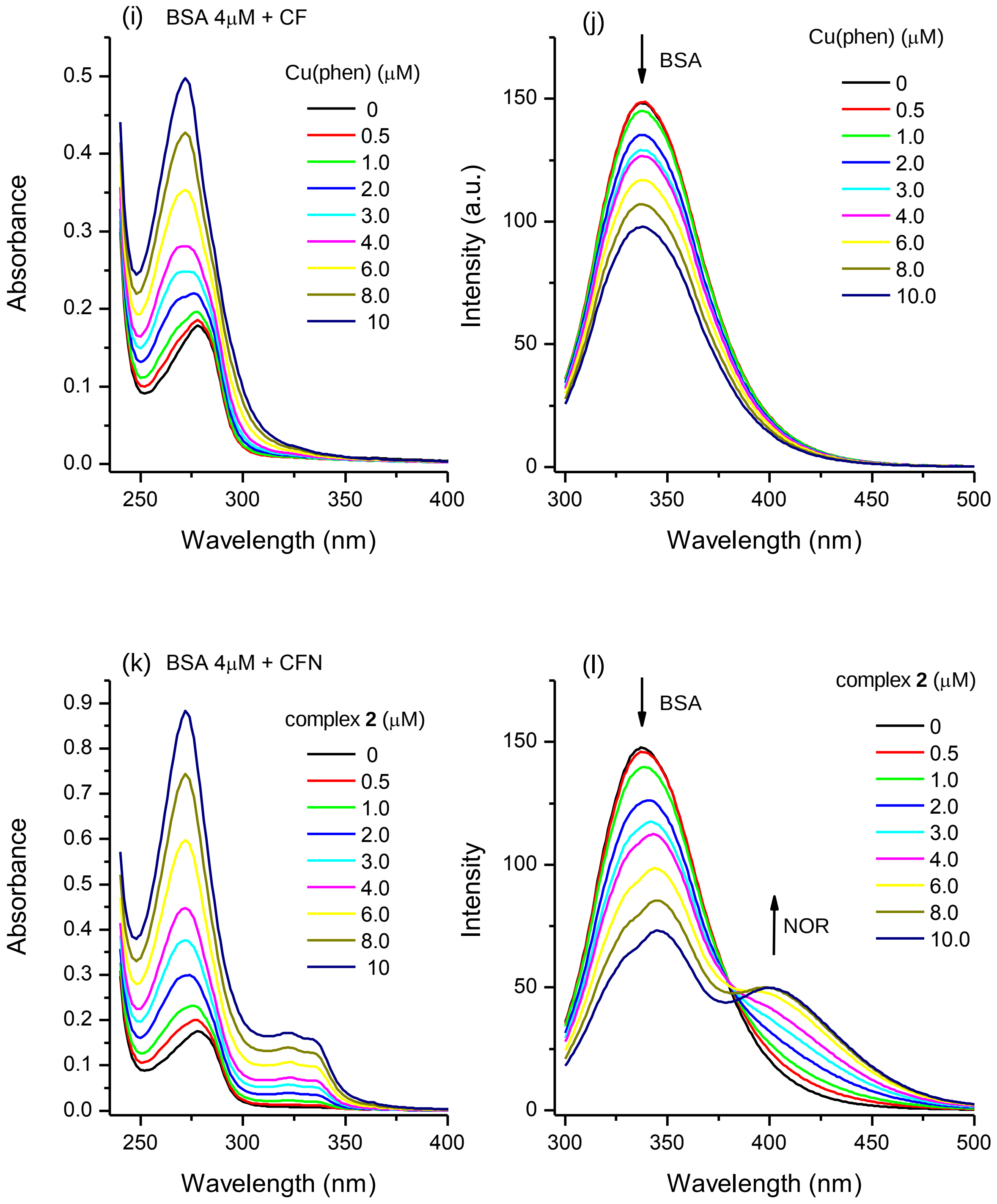

Figure S1. UV absorption (a, c, e, g, i, k) and raw fluorescence emission (b, d, f, h, j, l) spectra of BSA (4 μM) with increasing amounts of copper, norfloxacin and copper complexes. (a, b) norfloxacin; (c, d) copper(II); (e, f) Cu(bipy); (g, h) complex (1); (i, j) Cu(phen); (k, l) complex (2). The fluorescence peak around 400 nm is due to direct excitation of NOR dissociated from Cu(II).

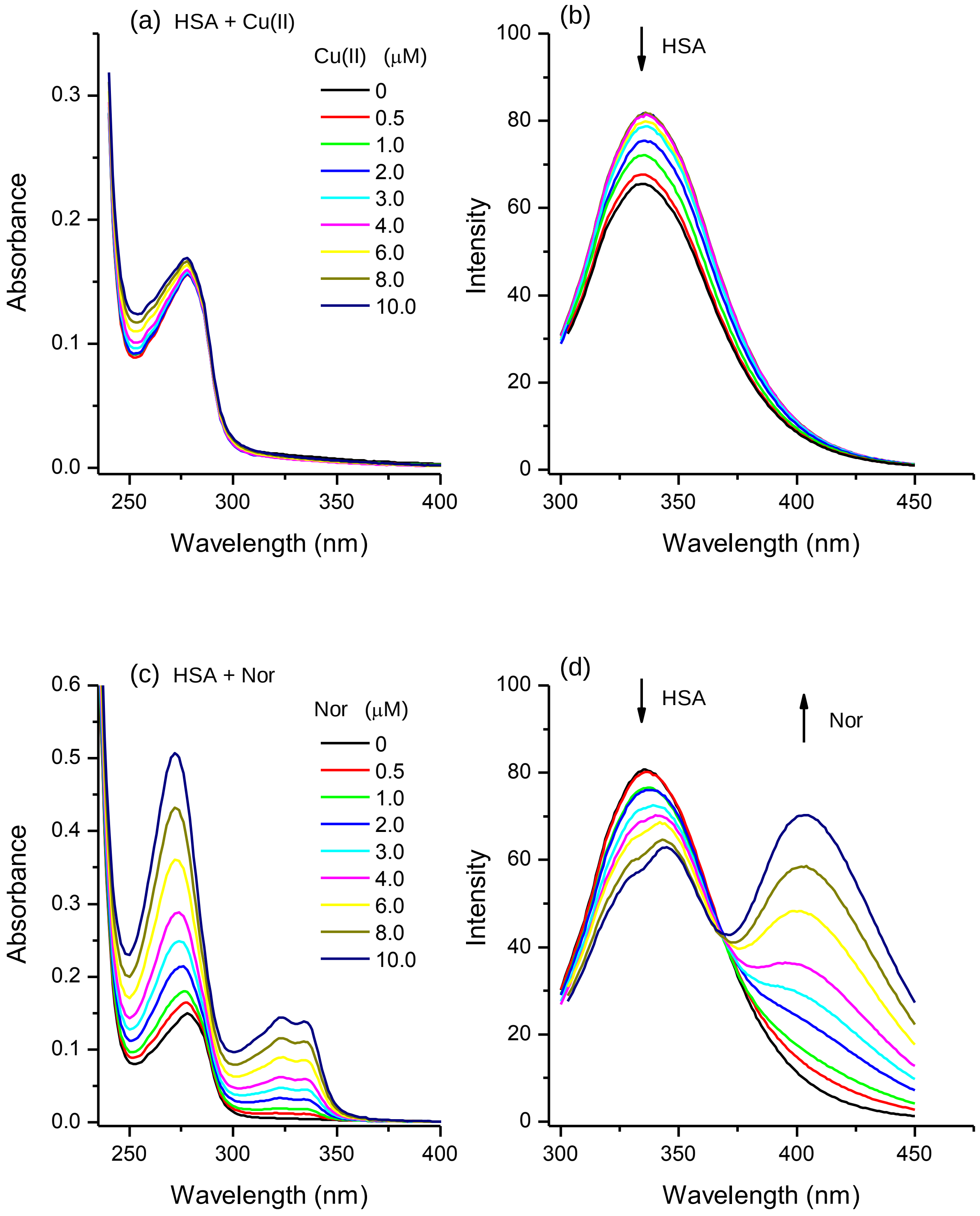

(a) HSA + Cu(II)
Cu(II) (μM)
0
0.5
1.0
2.0
3.0
4.0
6.0
8.0
10.0
Absorbance
Wavelength (nm)
(b)
HSA
Intensity
Wavelength (nm)
(c) HSA + Nor
Nor (μM)
0
0.5
1.0
2.0
3.0
4.0
6.0
8.0
10.0
Absorbance
Wavelength (nm)
(d)
HSA
Nor
Intensity
Wavelength (nm)

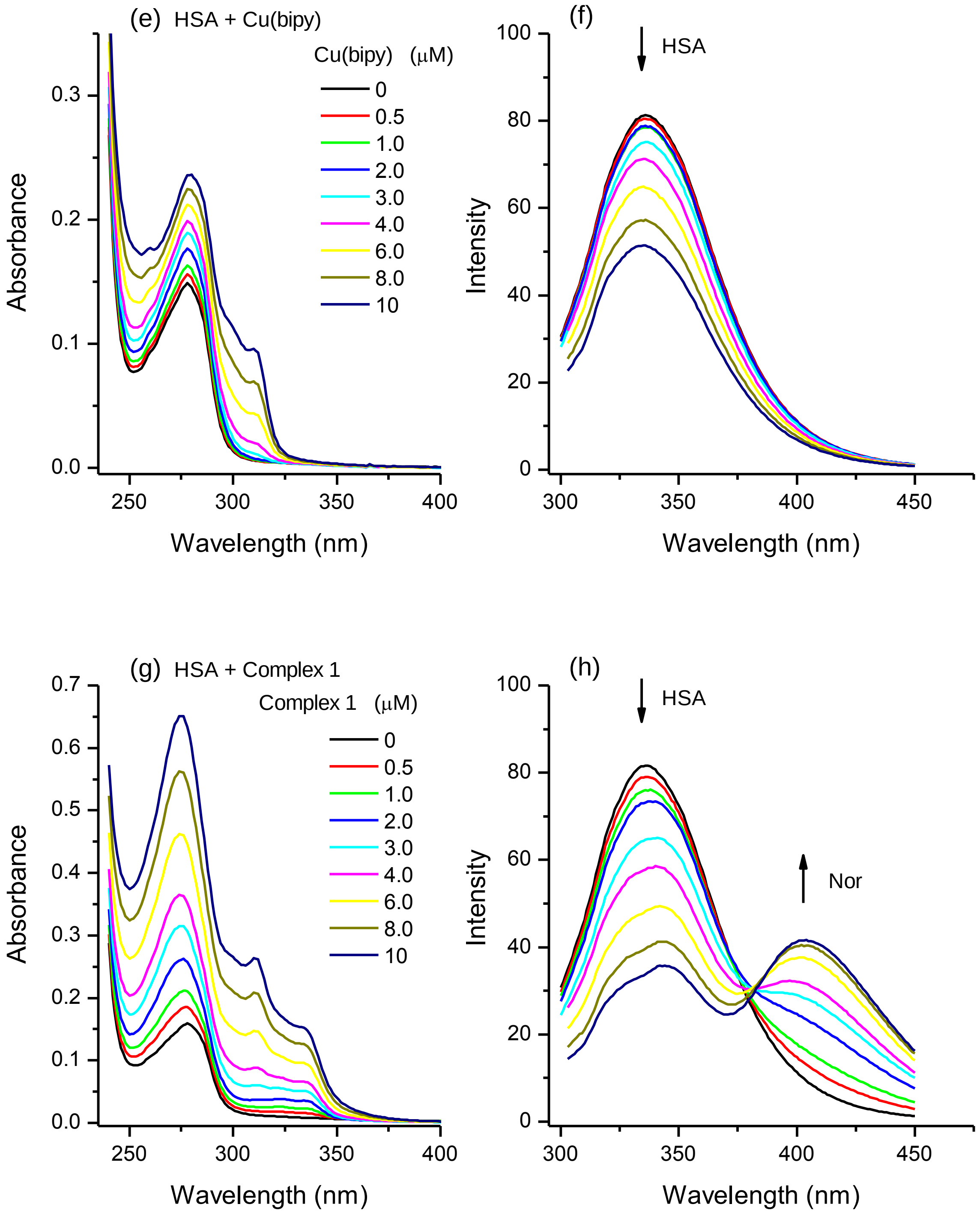
(e) HSA + Cu(bipy)
Cu(bipy) (μM)
0
0.5
1.0
2.0
3.0
4.0
6.0
8.0
10
Absorbance
Wavelength (nm)
(f)
HSA
Intensity
Wavelength (nm)
(g) HSA + Complex 1
Complex 1 (μM)
0
0.5
1.0
2.0
3.0
4.0
6.0
8.0
10
Absorbance
Wavelength (nm)
(h)
HSA
Nor
Intensity
Wavelength (nm)

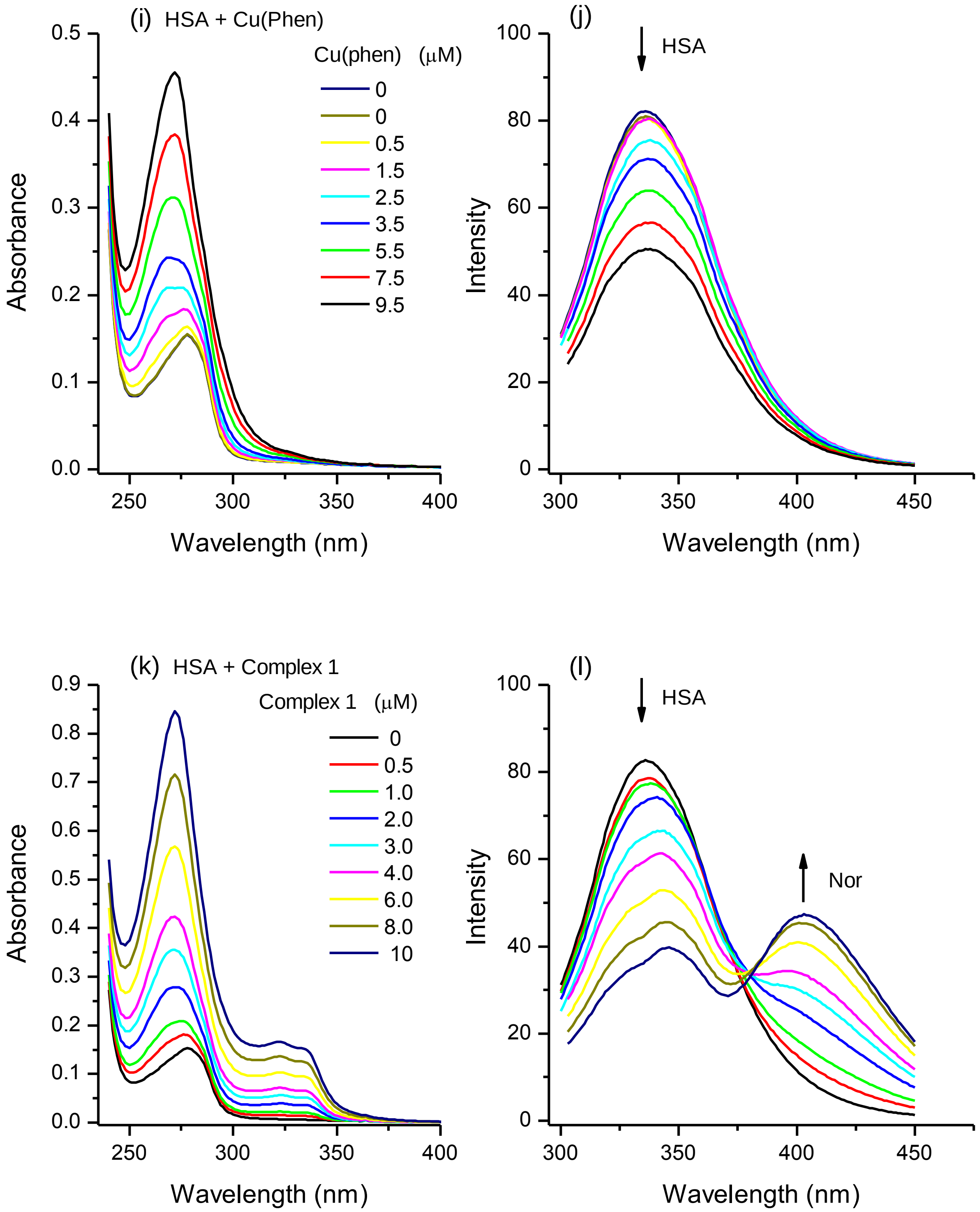

Figure S2. UV absorption (a, c, e, g, i, k) and raw fluorescence emission (b, d, f, h, j, l) spectra of HSA (4 μM) with increasing amounts of copper, norfloxacin and copper complexes. (a, b) norfloxacin; (c, d) copper(II); (e, f) Cu(bipy); (g, h) complex (1); (i, j) Cu(phen); (k, l) complex (2). The fluorescence peak around 400 nm is due to direct excitation of NOR dissociated from Cu(II).

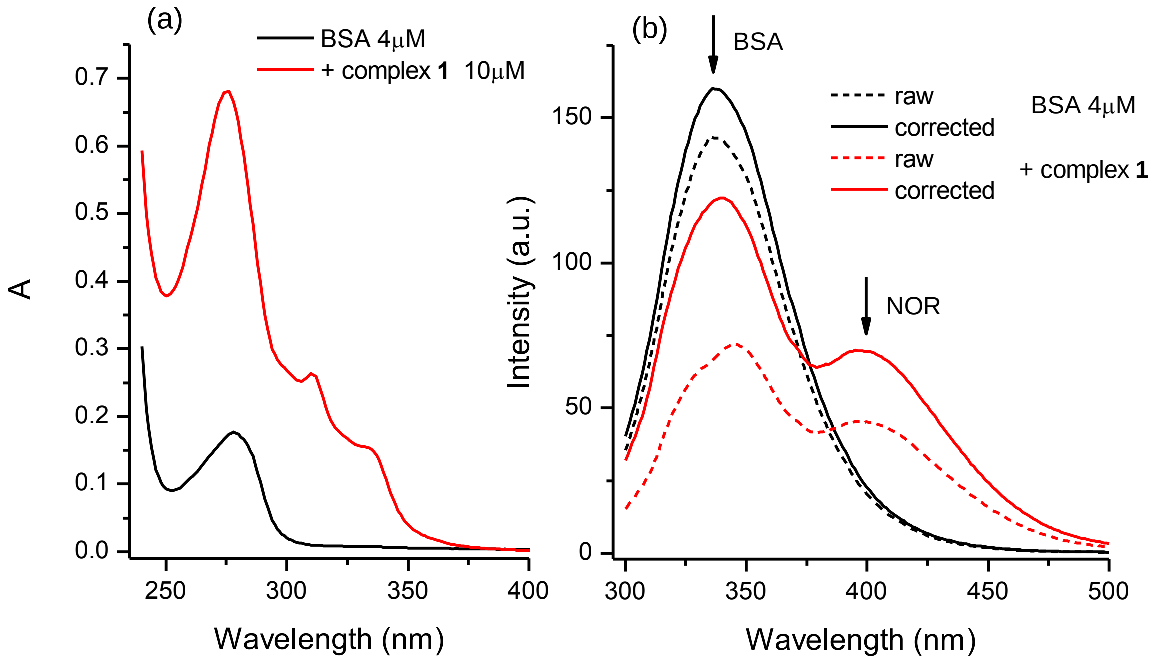

Figure S3. Correction of the fluorescence emission spectra. (a) UV absorption of BSA (4 μM) without (black) and with (red) complex 1, 10μM. These spectra were used to correct the raw fluorescence emission spectra (b, dashed lines) using the expression

$$F_{corr} = F_{obs}\, 10^{[(A_{ex}+A_{em})\ell/2]}$$

where F_{corr} and F_{obs} are the corrected and observed fluorescence intensity, A_{ex} and A_{em} are the absorbance at the excitation and emission wavelengths, respectively, and the optical path in cm, ℓ , is equal to 1. The corrected fluorescence emission spectra appear as solid lines in (b). Note that the fluorescence spectrum of BSA in the presence of complex **1** (red dashed line) not only has a lower intensity, but is also distorted because of complex **1** absorption in the region between 300 and 350 nm.

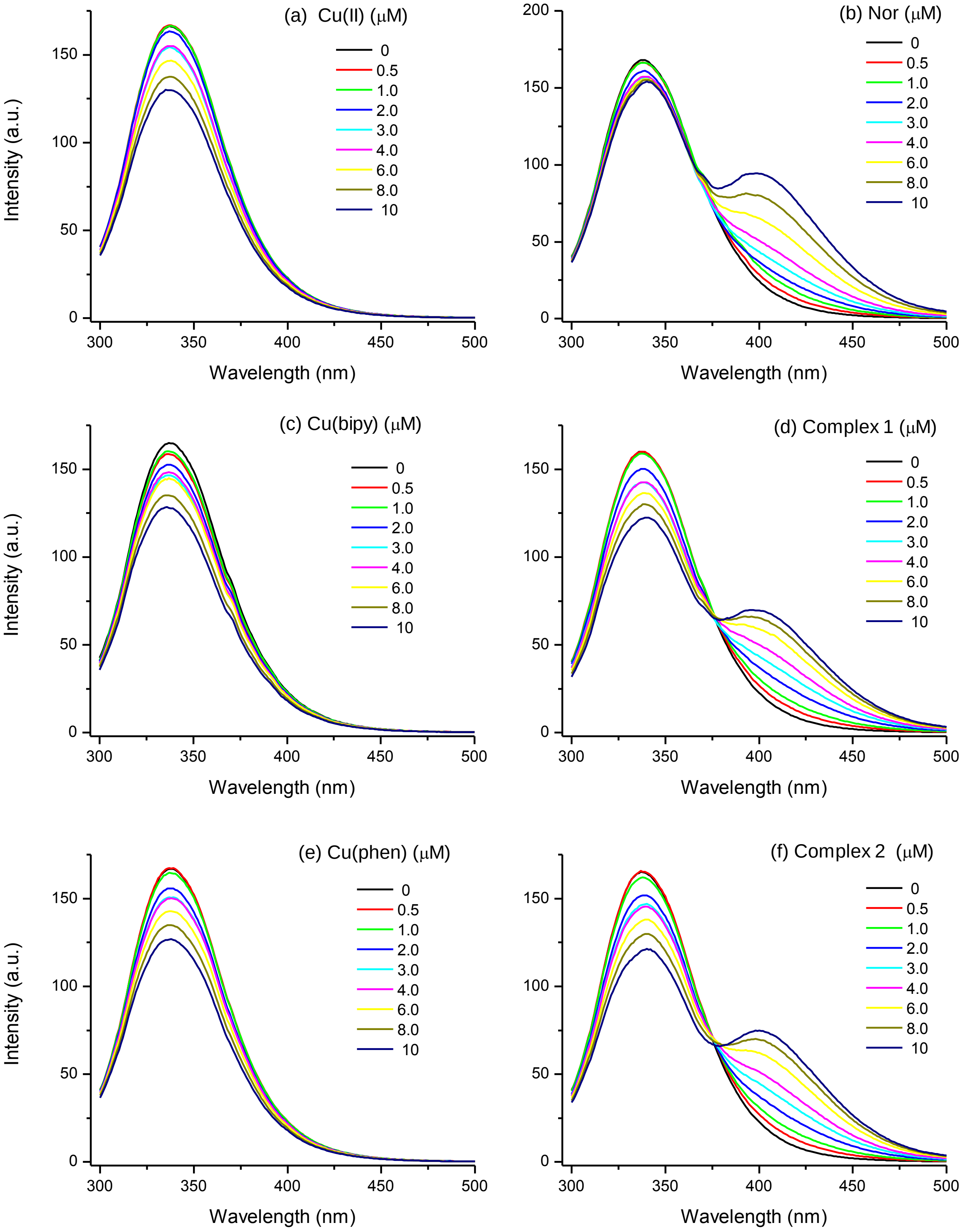

Figure S4. Corrected fluorescence emission spectra of BSA (4 μM) with increasing amounts of copper, norfloxacin or copper complexes. (a) copper(II); (b) norfloxacin; (c) Cu(bipy); (d) complex (1); (e) Cu(phen); (f) complex (2). The fluorescence peak around 400 nm is due to direct excitation of NOR dissociated from the Cu(II) complexes.

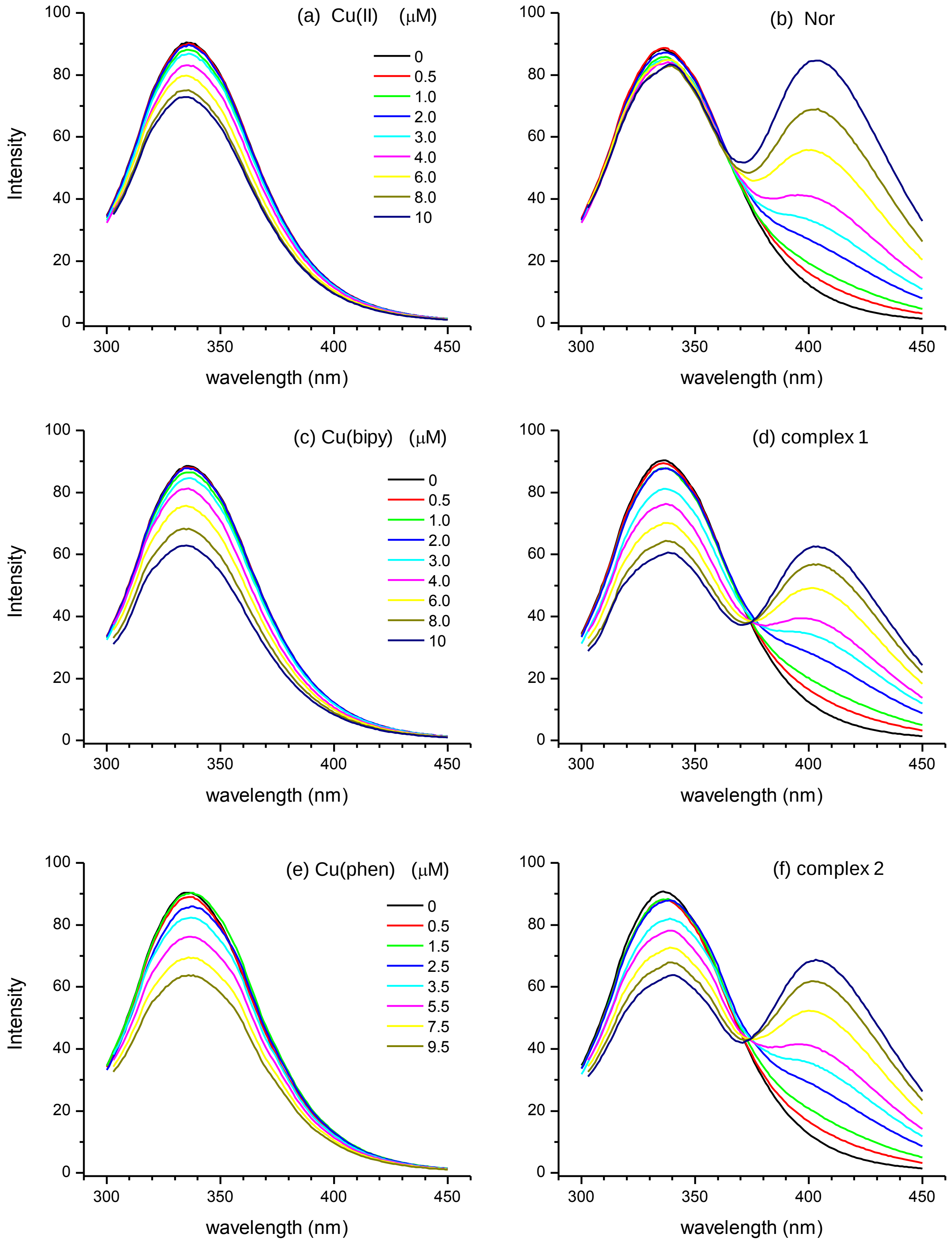

Figure S5. Corrected fluorescence emission spectra of HSA (4 μM) with increasing amounts of copper, norfloxacin or copper complexes. (a) copper(II); (b) norfloxacin; (c) Cu(bipy); (d) complex (1); (e) Cu(phen); (f) complex (2). The fluorescence peak around 400 nm is due to direct excitation of NOR dissociated from the Cu(II) complexes. Legends for the quencher concentrations are the same, except for (e).
